# Supplementary material for: Spatial transcriptomics reveals mechanism of autoimmunity driven by internalized autoantibodies
Source: medRxiv. 2026 Mar 5:2026.02.14.26346329. Originally published 2026 Feb 17. Preprint. [Version 2] doi: 10.64898/2026.02.14.26346329 (PMC12934838; doi:10.64898/2026.02.14.26346329)
Supplement: Supplement 2 [file media-2.pdf]

Supplementary Table 1. Differential expression of anti-Mi2-specific genes in muscle biopsies of anti-Mi2-positive patients and anti-PM/Scl-positive patients relative to all the other samples. Pos, position in the complete differential expression table; logFC, log_2 fold change; adj.P.Val, Benjamini-Hochberg-adjusted P value.

	Anti-Mi2 vs. all			Anti-PM/Scl vs. all		
Gene	Pos	logFC	adj.P.Val	Pos	logFC	adj.P.Val
PRR35	1	3.9	2.5e-32	20,640	0.4	4.3e-01
IFITM5	2	3.8	2.9e-32	36,321	-0.1	8.4e-01
SCRT1	3	4.1	5.0e-32	33,329	0.2	7.7e-01
P2RX2	5	3.8	6.9e-30	19,464	0.5	3.9e-01
DHRS2	11	3.4	2.8e-20	43,901	0.0	9.7e-01
CHRM4	13	2.9	5.8e-20	42,428	-0.1	9.4e-01
KCNJ4	16	3.2	1.8e-18	42,665	0.1	9.5e-01
COX6B2	18	3.0	2.3e-17	35,506	0.2	8.2e-01
RAB3B	23	3.0	1.0e-16	8,263	1.1	7.5e-02
ENSG00000289332.1	28	2.7	9.9e-15	41,843	0.1	9.3e-01
PLPPR3	31	2.7	3.8e-14	41,517	-0.1	9.3e-01
WFDC2	38	2.5	8.9e-13	27,373	-0.4	6.2e-01
TMEM151A	48	2.4	6.1e-12	33,711	0.2	7.8e-01
KIF1A	49	2.7	6.3e-12	23,004	-0.6	5.0e-01
ENSG00000255375.3	56	2.4	2.3e-11	36,872	0.1	8.5e-01
KREMEN2	69	2.8	3.4e-11	41,385	-0.1	9.3e-01
ANKRD20A19P	95	2.4	2.6e-10	20,493	0.5	4.2e-01
COL2A1	149	2.2	3.6e-09	42,905	0.1	9.5e-01
ECE2	161	2.1	4.5e-09	26,432	0.3	6.0e-01
IGLON5	220	2.2	1.5e-08	6,184	1.0	3.9e-02
FAM171A2	230	1.7	1.6e-08	20,374	0.4	4.2e-01
CAMKV	243	2.1	1.8e-08	10,673	0.8	1.3e-01
CLDN6	365	2.1	8.9e-08	44,856	-0.0	9.8e-01
ENSG00000286311.1	407	1.9	1.2e-07	35,885	0.2	8.3e-01
FBXL16	416	2.0	1.3e-07	34,888	-0.2	8.1e-01
B4GALNT4	476	1.8	2.5e-07	40,237	-0.1	9.1e-01
TMEM151B	522	1.9	3.9e-07	7,772	0.9	6.5e-02
ENSG00000251511.1	533	1.5	4.2e-07	31,396	0.2	7.3e-01
PTH2	560	1.4	4.9e-07	41,308	0.1	9.3e-01
CPNE6	575	2.1	5.5e-07	24,094	0.4	5.3e-01
SHISA7	755	2.0	1.5e-06	27,415	0.3	6.2e-01
LKAAEAR1	764	1.7	1.6e-06	17,248	0.5	3.2e-01
GIMD1	789	1.4	1.9e-06	9,779	0.6	1.1e-01
NKAIN4	853	2.1	2.5e-06	37,213	-0.2	8.5e-01
GJD2	902	2.0	3.1e-06	18,502	-0.6	3.6e-01
GCGR	931	2.1	3.5e-06	22,377	0.5	4.8e-01
PRKCG	968	1.8	4.1e-06	16,296	0.6	2.9e-01
ENSG00000256481.1	1,002	1.6	4.8e-06	33,689	0.2	7.8e-01
BANCR	1,053	1.6	6.1e-06	30,304	0.2	7.0e-01
MMEL1-AS1	1,148	1.3	8.4e-06	22,848	0.3	4.9e-01
OVOL1	1,330	1.7	1.5e-05	33,390	0.2	7.7e-01
SMIM24	1,379	1.7	1.7e-05	29,643	0.3	6.8e-01
TNNI3	1,646	1.4	3.4e-05	20,297	-0.5	4.2e-01
HCN2	1,763	1.5	4.5e-05	9,100	0.8	9.2e-02
ALPG	1,851	1.8	5.5e-05	1,302	1.8	4.2e-04
CT69	1,918	1.5	6.4e-05	27,704	-0.3	6.3e-01
ENSG00000142539.9	1,966	2.0	6.8e-05	4,367	1.5	1.6e-02
C5ORF47	2,152	1.4	9.8e-05	39,707	-0.1	9.0e-01
CACNA1G	2,183	1.3	1.1e-04	24,912	-0.4	5.5e-01
TEX19	2,207	1.5	1.1e-04	38,342	0.1	8.7e-01
IL11	2,233	1.5	1.2e-04	22,338	0.4	4.8e-01
COL26A1	2,238	1.5	1.2e-04	9,059	0.9	9.1e-02
FAM131C	2,304	1.5	1.4e-04	25,062	-0.4	5.6e-01
ESPN	2,461	1.5	1.8e-04	16,727	0.6	3.0e-01
CHGA	2,679	1.5	2.6e-04	26,193	0.3	5.9e-01
LINC02066	2,714	1.2	2.7e-04	16,800	0.5	3.1e-01
ENSG00000251076.1	2,778	1.7	3.0e-04	26,879	-0.4	6.1e-01
UOX	2,811	1.3	3.1e-04	31,473	-0.2	7.3e-01

	Anti-Mi2 vs. all			Anti-PM/Scl vs. all		
**Gene**	**Pos**	**logFC**	**adj.P.Val**	**Pos**	**logFC**	**adj.P.Val**
**LINC00624**	3,044	1.3	4.3e-04	37,463	-0.1	8.6e-01
**ENTPD8**	3,237	1.4	5.7e-04	24,051	0.4	5.3e-01
**TH**	3,347	1.3	6.5e-04	36,567	0.1	8.4e-01
**SSU72P8**	3,383	1.1	6.8e-04	1,539	1.3	7.9e-04
**RAC3**	3,573	1.1	8.5e-04	20,171	0.4	4.2e-01
**RAP1GAP**	3,660	1.2	9.3e-04	13,683	-0.7	2.1e-01
**KLC3**	3,767	1.3	1.0e-03	31,908	0.3	7.4e-01
**CAPN12**	3,929	1.1	1.2e-03	43,666	-0.0	9.6e-01
**C1QTNF8**	4,174	1.3	1.5e-03	23,133	0.4	5.0e-01
**GRIN2D**	4,331	1.1	1.7e-03	34,095	-0.2	7.9e-01
**KCNQ2**	5,061	1.3	3.2e-03	38,536	-0.1	8.8e-01
**CTSV**	5,241	0.7	3.6e-03	14,994	-0.4	2.5e-01
**GRIN3B**	5,546	1.2	4.6e-03	3,309	1.3	7.7e-03
**FLRT1**	5,935	1.1	6.1e-03	45,852	0.0	1.0e+00
**PALM3**	5,979	1.0	6.3e-03	26,955	-0.3	6.1e-01
**JPH3**	6,304	1.0	7.9e-03	42,715	-0.1	9.5e-01
**SPIB**	6,580	0.8	9.4e-03	4,060	0.9	1.4e-02
**CAMSAP3**	6,687	1.1	1.0e-02	38,196	0.1	8.7e-01
**UTF1**	6,805	0.9	1.1e-02	37,411	0.1	8.6e-01
**PPP1R1B**	6,881	1.4	1.1e-02	11,735	-1.2	1.6e-01
**CBARP**	6,888	0.7	1.1e-02	10,642	0.6	1.3e-01
**CASKIN1**	7,086	0.9	1.3e-02	23,155	-0.4	5.0e-01
**HPCA**	7,287	0.9	1.4e-02	17,836	0.5	3.4e-01
**SEMA4G**	7,448	0.6	1.5e-02	4,317	-0.8	1.6e-02
**LGI3**	8,003	1.0	2.0e-02	19,788	-0.5	4.0e-01
**ENSG00000218416.4**	8,066	0.9	2.0e-02	45,289	0.0	9.9e-01
**AQP5**	8,729	0.9	2.7e-02	21,512	0.4	4.5e-01
**SMPD4P1**	8,854	1.0	2.8e-02	34,461	0.2	8.0e-01
**CACNA1I**	8,931	0.9	2.9e-02	38,365	0.1	8.7e-01
**ENSG00000260293.2**	9,144	0.8	3.2e-02	15,413	0.5	2.7e-01
**CRB3**	9,914	0.8	4.4e-02	45,033	-0.0	9.9e-01
**MSI1**	10,015	0.8	4.5e-02	29,875	0.3	6.9e-01
**PDIA2**	10,063	0.8	4.6e-02	15,196	-0.6	2.6e-01
**MADCAM1**	10,489	0.6	5.3e-02	10,038	0.6	1.2e-01
**VWA5B2**	10,720	0.8	5.7e-02	19,911	0.5	4.1e-01
**STAC2**	12,426	0.7	9.5e-02	8,663	0.8	8.3e-02
**ARHGDIG**	12,727	0.8	1.0e-01	10,228	-0.9	1.2e-01
**SLC29A4**	13,376	0.6	1.2e-01	4,157	-1.3	1.5e-02
**ENSG00000275437.1**	13,527	0.6	1.2e-01	7,970	0.8	6.9e-02
**PRKAR1B**	15,145	0.3	1.7e-01	13,091	0.3	2.0e-01
**KBTBD11-AS1**	16,067	0.5	2.1e-01	41,669	0.1	9.3e-01
**ABCG4**	18,233	0.4	2.9e-01	21,429	0.4	4.5e-01
**EFNA3**	19,086	-0.4	3.3e-01	27,852	-0.2	6.3e-01
**TMEM145**	19,372	0.4	3.4e-01	9,193	-0.7	9.5e-02
**BAIAP3**	25,949	0.2	6.3e-01	5,807	-0.7	3.4e-02
**ENSG00000267892.1**	26,756	0.2	6.6e-01	20,577	0.3	4.3e-01
**ENSG00000223561.7**	29,623	0.2	7.7e-01	7,522	-0.9	6.1e-02
**RAB26**	31,400	-0.2	8.2e-01	26,383	-0.3	6.0e-01
**DACT3**	31,684	-0.1	8.3e-01	19,613	0.3	4.0e-01
**YBX2**	35,516	0.1	9.2e-01	33,614	-0.1	7.8e-01
**SOX15**	38,072	-0.1	9.5e-01	4,889	-1.0	2.2e-02
**ENSG00000169093.16**	38,229	0.0	9.5e-01	16,785	-0.3	3.1e-01
**DLGAP3**	39,181	0.1	9.6e-01	19,453	0.4	3.9e-01
**ZNF467**	40,148	-0.0	9.7e-01	45,843	0.0	1.0e+00
**CADM4**	40,341	0.0	9.7e-01	8,798	-0.5	8.5e-02

Supplementary Table 2. Differential expression of anti-PM/Scl-specific genes (PMID 38902010) in muscle biopsies of anti-PM/Scl-positive patients and anti-Mi2-positive patients relative to all the other samples. Pos, position in the complete differential expression table; logFC, $\log_2$ fold change; adj.P.Val, Benjamini-Hochberg-adjusted P value.

	Anti-PM/Scl vs. all			Anti-Mi2 vs. all		
**Gene**	**Pos**	**logFC**	**adj.P.Val**	**Pos**	**logFC**	**adj.P.Val**
**ENSG00000268403.2**	1	2.6	2.2e-33	740	-1.5	1.4e-06
**ENSG00000276216.1**	2	4.5	1.3e-31	12,611	-0.8	9.9e-02
**ENSG00000289412.1**	3	2.7	4.4e-31	2,557	-1.3	2.1e-04
**ENSG00000288879.1**	4	3.6	7.6e-26	43,206	-0.0	9.9e-01
**ENSG00000288751.1**	5	3.7	3.3e-25	25,338	-0.3	6.1e-01
**ENSG00000287584.1**	6	3.5	2.1e-24	19,495	-0.4	3.4e-01
**ENSG00000226380.9**	7	2.7	2.6e-23	36,171	-0.1	9.3e-01
**ENSG00000289296.1**	8	3.4	2.4e-22	10,028	-0.7	4.6e-02
**MIR378D2HG**	9	3.3	1.7e-21	20,814	-0.4	4.0e-01
**ENSG00000288988.1**	10	2.7	2.6e-21	14,972	-0.6	1.7e-01
**ENSG00000261334.1**	11	3.5	1.8e-20	44,744	0.0	9.9e-01
**ENSG00000289030.1**	12	3.3	4.9e-20	12,825	-0.6	1.0e-01
**ENSG00000264739.1**	13	3.4	1.1e-18	32,691	-0.2	8.5e-01
**ENSG00000273210.1**	14	2.4	1.8e-18	24,175	0.3	5.5e-01
**ENSG00000289341.1**	15	3.3	2.3e-18	16,718	-0.6	2.3e-01
**ENSG00000288943.1**	16	3.5	6.1e-18	14,566	-0.6	1.6e-01
**ENSG00000289103.1**	17	3.1	1.6e-17	4,136	-1.1	1.5e-03
**ENSG00000289499.1**	18	3.0	2.7e-17	12,500	-0.6	9.6e-02
**ENSG00000288900.1**	19	3.3	5.3e-17	17,660	-0.5	2.7e-01
**ENSG00000234432.4**	20	2.5	5.5e-17	13,187	-0.7	1.1e-01
**ENSG00000288865.1**	21	2.9	1.1e-16	28,333	-0.2	7.2e-01
**ENSG00000287979.1**	23	3.4	1.1e-16	17,226	-0.7	2.5e-01
**ENSG00000239705.2**	25	2.8	1.6e-16	25,954	-0.2	6.3e-01
**ENSG00000289221.1**	26	2.9	2.0e-16	24,269	-0.3	5.6e-01
**ENSG00000289115.1**	27	2.8	2.2e-16	26,280	-0.2	6.4e-01
**ENSG00000272967.1**	28	2.8	8.0e-16	17,163	-0.5	2.5e-01
**TRIM8-DT**	29	2.0	1.2e-15	4,792	-1.0	2.6e-03
**BMP2K-DT**	30	2.8	7.8e-15	5,770	-1.2	5.4e-03
**ENSG00000288919.1**	31	2.7	9.5e-15	2,131	-1.7	9.5e-05
**LINC01126**	32	2.4	1.6e-14	9,080	-0.8	3.1e-02
**TIMMDC1-DT**	33	2.7	1.6e-14	28,667	-0.2	7.3e-01
**ENSG00000260369.2**	34	2.7	2.4e-14	14,839	-0.6	1.7e-01
**ENSG00000272719.1**	35	2.7	4.7e-14	19,904	-0.4	3.6e-01
**ENSG00000286409.2**	36	2.7	4.7e-14	13,122	-0.5	1.1e-01
**ENSG00000279259.1**	37	2.4	5.0e-14	7,544	-0.9	1.6e-02
**ENSG00000288955.1**	38	2.7	5.7e-14	20,880	-0.3	4.0e-01
**ENSG00000287654.1**	39	2.6	6.3e-14	25,537	-0.3	6.1e-01
**ENSG00000289200.1**	40	2.7	4.2e-13	40,881	-0.0	9.7e-01
**ENSG00000265100.1**	41	2.6	1.3e-12	15,645	-0.6	1.9e-01
**UAP1-DT**	42	2.4	1.3e-12	27,735	-0.2	7.0e-01
**ENSG00000289202.1**	43	2.7	2.0e-12	19,159	-0.4	3.3e-01
**ENSG00000288804.1**	45	2.5	2.4e-12	16,181	-0.5	2.1e-01
**ENSG00000289044.1**	46	2.7	2.6e-12	14,130	-0.5	1.4e-01
**ENSG00000289142.1**	47	2.2	3.8e-12	23,540	-0.3	5.2e-01
**ENSG00000288996.1**	48	2.4	3.9e-12	31,325	-0.2	8.2e-01
**ENSG00000289288.1**	49	2.6	4.2e-12	32,002	0.2	8.4e-01
**MIR23AHG**	50	1.2	4.5e-12	11,078	-0.4	6.4e-02
**ENSG00000289152.1**	52	2.5	6.1e-12	34,381	-0.1	9.0e-01
**ENSG00000286408.1**	53	1.9	6.2e-12	3,172	-1.1	5.2e-04
**TTC32-DT**	54	2.5	6.3e-12	17,194	-0.4	2.5e-01
**ENSG00000289379.1**	55	2.6	7.5e-12	31,827	-0.1	8.3e-01

	Anti-PM/Scl vs. all			Anti-Mi2 vs. all		
**Gene**	**Pos**	**logFC**	**adj.P.Val**	**Pos**	**logFC**	**adj.P.Val**
**ENSG00000288963.1**	56	2.5	1.1e-11	22,085	-0.3	4.6e-01
**ENSG00000279212.1**	57	2.3	1.2e-11	11,772	-0.7	7.9e-02
**ENSG00000289159.1**	58	2.3	1.4e-11	2,449	-1.5	1.7e-04
**ENSG00000289154.1**	59	1.7	1.6e-11	3,454	-1.0	7.4e-04
**ENSG00000288872.1**	61	2.4	1.9e-11	21,618	-0.3	4.4e-01
**ENSG00000289551.1**	62	2.4	3.2e-11	31,639	-0.1	8.3e-01
**LINC00677**	63	2.3	3.8e-11	44,449	0.0	9.9e-01
**TNFAIP8L1**	64	1.0	3.8e-11	26,390	0.1	6.5e-01
**ENSG00000270019.1**	66	2.6	5.9e-11	37,236	-0.1	9.4e-01
**ENSG00000289055.1**	68	2.5	1.4e-10	21,962	-0.3	4.5e-01
**ENSG00000289303.1**	70	2.4	1.8e-10	32,361	-0.1	8.5e-01
**SLC38A2-AS1**	71	2.4	1.8e-10	40,653	-0.0	9.7e-01
**ENSG00000261242.1**	72	2.3	2.4e-10	28,825	-0.2	7.4e-01
**THOC1-DT**	73	1.9	2.4e-10	2,959	-1.1	3.8e-04
**TERC**	74	2.7	3.9e-10	33,904	-0.1	8.9e-01
**ENSG00000289235.1**	75	2.3	5.0e-10	34,172	-0.1	8.9e-01
**SPACA6P-AS**	76	2.5	6.3e-10	11,328	-0.7	6.9e-02
**ENSG00000289478.1**	77	2.6	7.8e-10	28,259	-0.2	7.2e-01
**ENSG00000287821.1**	79	2.1	9.7e-10	35,055	0.1	9.1e-01
**ENSG00000245651.3**	80	2.3	9.9e-10	14,183	-0.6	1.4e-01
**ENSG00000241666.2**	82	2.2	1.6e-09	20,379	-0.4	3.8e-01
**ENSG00000286113.1**	85	2.4	2.2e-09	41,761	-0.0	9.8e-01
**ENSG00000288939.1**	87	2.3	2.3e-09	16,391	-0.5	2.2e-01
**ENSG00000289550.1**	88	2.3	2.6e-09	16,091	-0.6	2.1e-01
**ENSG00000224505.3**	89	1.4	2.7e-09	609	-1.4	6.9e-07
**ENSG00000284484.1**	90	2.7	3.0e-09	12,957	0.8	1.1e-01
**ENSG00000288744.1**	92	2.3	3.5e-09	21,588	-0.3	4.3e-01
**RNVU1-14**	93	2.4	3.9e-09	36,863	-0.1	9.4e-01
**ENSG00000255089.1**	97	2.3	4.3e-09	18,671	-0.4	3.1e-01
**MSRA-DT**	101	2.3	7.5e-09	13,762	-0.6	1.3e-01
**ENSG00000286577.1**	104	2.1	9.6e-09	32,344	-0.1	8.5e-01
**ENSG00000289317.1**	106	2.0	1.0e-08	27,881	-0.2	7.0e-01
**ENSG00000272768.1**	107	2.0	1.1e-08	17,274	0.5	2.5e-01
**ENSG00000289506.1**	109	2.0	1.2e-08	25,578	-0.3	6.2e-01
**ENSG00000284602.1**	110	1.4	1.2e-08	4,042	-0.9	1.4e-03
**ENSG00000272426.1**	111	2.2	1.3e-08	16,711	-0.5	2.3e-01
**ENSG00000274213.1**	112	2.1	1.3e-08	14,195	-0.7	1.4e-01
**ENSG00000282936.2**	113	2.3	1.5e-08	8,511	-0.9	2.4e-02
**TRIM51BP**	114	2.6	1.6e-08	21,559	0.4	4.3e-01
**ENSG00000255647.3**	115	2.2	1.6e-08	41,156	-0.0	9.7e-01
**ENSG00000288842.1**	119	2.1	1.9e-08	19,144	-0.4	3.3e-01
**ENSG00000272953.1**	121	1.6	3.0e-08	1,290	-1.4	1.3e-05
**MIR5188**	122	1.9	3.1e-08	45,085	-0.0	1.0e+00
**ENSG00000273338.1**	125	2.6	3.6e-08	19,582	-0.5	3.4e-01
**ENSG00000253838.1**	127	2.1	3.6e-08	29,758	0.2	7.7e-01
**ENSG00000259135.1**	129	2.2	3.8e-08	9,358	-0.8	3.5e-02
**SMG7-AS1**	131	2.0	3.9e-08	13,052	-0.6	1.1e-01
**PRAMEF13**	133	2.4	4.1e-08	26,978	0.3	6.7e-01
**ENSG00000288929.1**	134	2.4	4.6e-08	11,545	-0.7	7.3e-02
**FSCN1**	139	1.3	5.3e-08	369	1.2	9.0e-08
**RABEP2**	140	0.8	6.1e-08	42,537	0.0	9.8e-01
**DDX39B-AS1**	141	2.0	6.3e-08	19,955	-0.4	3.6e-01
**ENSG00000278743.1**	142	1.9	6.4e-08	23,365	-0.4	5.1e-01
**LINC01089**	153	0.8	1.1e-07	19,113	-0.2	3.3e-01
**MIRLET7BHG**	164	1.2	1.4e-07	2,798	-1.0	3.1e-04
**ENSG00000287070.1**	166	2.3	1.5e-07	11,854	-0.8	8.1e-02
**ENSG00000288866.1**	167	2.1	1.5e-07	30,386	-0.2	7.9e-01

	Anti-PM/Scl vs. all			Anti-Mi2 vs. all		
**Gene**	**Pos**	**logFC**	**adj.P.Val**	**Pos**	**logFC**	**adj.P.Val**
**ENSG00000286444.1**	168	2.0	1.6e-07	35,673	0.1	9.2e-01
**ENSG00000274751.1**	173	1.7	1.9e-07	6,863	-0.9	1.1e-02
**ENSG00000289177.1**	177	2.1	2.0e-07	35,133	-0.1	9.1e-01
**ENSG00000289229.1**	179	2.0	2.0e-07	40,050	-0.1	9.7e-01
**ENSG00000289182.1**	180	1.5	2.1e-07	16,272	-0.5	2.1e-01
**ENSG00000288896.1**	181	2.0	2.1e-07	21,197	-0.4	4.2e-01
**ENSG00000276524.1**	182	1.9	2.2e-07	12,585	-0.6	9.9e-02
**LINC01424**	184	1.7	2.4e-07	12,310	-0.6	9.2e-02
**ENSG00000268670.1**	190	2.0	2.5e-07	11,800	-0.7	8.0e-02
**ENSG00000254028.1**	191	2.0	2.6e-07	16,406	-0.4	2.2e-01
**CAPN10-DT**	196	1.3	2.8e-07	661	-1.4	8.9e-07
**RENO1**	198	1.1	2.8e-07	492	-1.3	2.8e-07
**ENSG00000289257.1**	207	1.9	3.5e-07	40,249	0.0	9.7e-01
**AFF4-DT**	211	1.9	3.6e-07	35,793	0.1	9.2e-01
**PRND**	212	2.4	3.6e-07	7,192	-1.4	1.3e-02
**ENSG00000286482.1**	222	1.7	4.6e-07	7,773	-0.8	1.7e-02
**LINC00896**	224	2.0	4.6e-07	18,577	-0.4	3.0e-01
**ENSG00000289457.1**	229	2.0	5.0e-07	5,073	-1.0	3.2e-03
**ENSG00000279491.1**	231	1.7	5.1e-07	1,916	-1.4	6.3e-05
**ENSG00000273064.1**	232	1.6	5.2e-07	12,614	-0.6	9.9e-02
**ENSG00000287697.1**	236	1.8	5.7e-07	18,920	-0.4	3.2e-01
**CPEB2-DT**	253	2.0	8.1e-07	40,730	-0.0	9.7e-01
**ENSG00000289005.1**	259	1.6	9.3e-07	4,528	-1.0	2.0e-03
**ENSG00000288813.1**	271	1.9	1.2e-06	33,664	-0.1	8.8e-01
**LINC02776**	272	1.9	1.2e-06	44,036	0.0	9.9e-01
**ENSG00000278158.1**	276	1.9	1.4e-06	16,987	-0.4	2.4e-01
**SDR42E2**	281	2.0	1.5e-06	25,980	-0.3	6.3e-01
**ENSG00000286881.1**	285	1.9	1.6e-06	22,759	-0.3	4.9e-01
**ENSG00000283959.2**	295	1.7	1.9e-06	17,958	-0.5	2.8e-01
**ANKH-DT**	296	1.8	2.0e-06	8,322	-0.8	2.2e-02
**COL18A1**	310	0.9	2.5e-06	21,567	0.2	4.3e-01
**KLF2-DT**	311	1.9	2.6e-06	34,117	-0.1	8.9e-01
**CAGE1**	315	2.0	2.6e-06	26,017	-0.3	6.3e-01
**ENSG00000272948.2**	320	1.6	2.7e-06	14,695	-0.6	1.6e-01
**ENSG00000274292.1**	333	1.7	3.2e-06	16,350	-0.6	2.2e-01
**ATXN7L3-AS1**	334	1.9	3.2e-06	25,528	-0.3	6.1e-01
**ENSG00000276744.1**	336	1.7	3.2e-06	12,715	-0.6	1.0e-01
**ENSG00000289626.1**	355	1.2	4.3e-06	4,481	-0.9	1.9e-03
**CLMAT3**	369	1.8	5.1e-06	16,032	-0.4	2.1e-01
**ENSG00000257258.2**	374	1.7	5.5e-06	24,579	0.3	5.7e-01
**ENSG00000278002.1**	379	1.6	6.0e-06	1,909	-1.4	6.2e-05
**ENSG00000273363.1**	382	1.7	6.1e-06	19,579	-0.4	3.4e-01
**ENSG00000269399.2**	384	1.4	6.2e-06	1,338	-1.4	1.5e-05
**ENSG00000287547.1**	393	1.6	7.2e-06	33,432	-0.1	8.7e-01
**ENSG00000289637.1**	403	2.0	7.7e-06	12,180	-0.6	8.8e-02
**ENSG00000289301.1**	431	1.7	9.4e-06	18,184	-0.4	2.9e-01
**ENSG00000289059.1**	436	1.7	9.6e-06	14,603	-0.6	1.6e-01
**ENSG00000260651.1**	453	1.8	1.2e-05	14,899	-0.5	1.7e-01
**ENSG00000288835.1**	484	1.7	1.5e-05	33,600	-0.1	8.8e-01
**ENSG00000289547.1**	488	1.9	1.6e-05	27,793	0.3	7.0e-01
**ENSG00000275709.1**	502	1.7	1.8e-05	39,268	0.1	9.6e-01
**MIR1915HG**	503	1.3	1.8e-05	11,042	-0.7	6.3e-02
**ENSG00000289031.1**	504	1.7	1.8e-05	17,651	-0.4	2.7e-01
**ENSG00000225945.1**	509	1.6	1.8e-05	39,336	0.1	9.6e-01
**ENSG00000278017.1**	545	1.6	2.3e-05	4,406	-1.1	1.8e-03
**ENSG00000289230.1**	550	1.8	2.4e-05	15,081	-0.6	1.7e-01
**ENSG00000230695.2**	564	1.7	2.6e-05	35,010	-0.1	9.1e-01

	Anti-PM/Scl vs. all			Anti-Mi2 vs. all		
**Gene**	**Pos**	**logFC**	**adj.P.Val**	**Pos**	**logFC**	**adj.P.Val**
**ENSG00000280035.1**	635	1.7	3.9e-05	22,528	-0.3	4.8e-01
**ENSG00000288746.1**	647	1.4	4.2e-05	24,187	-0.3	5.5e-01
**EGFL7**	662	0.8	4.5e-05	35,119	0.1	9.1e-01
**ENSG00000274251.1**	663	1.7	4.5e-05	25,787	-0.2	6.2e-01
**ENSG00000279140.1**	667	1.4	4.6e-05	7,790	-0.9	1.8e-02
**ENSG00000278932.5**	676	1.2	4.8e-05	1,648	-1.4	3.5e-05
**ENSG00000277020.4**	710	1.4	5.5e-05	5,238	-1.0	3.6e-03
**ENSG00000274737.1**	719	1.6	5.7e-05	17,000	-0.5	2.4e-01
**ENSG00000270012.1**	720	1.0	5.8e-05	3,427	-0.9	7.2e-04
**RN7SL521P**	723	1.6	6.0e-05	12,768	-0.6	1.0e-01
**ENSG00000288927.1**	728	1.6	6.3e-05	17,550	-0.5	2.6e-01
**ENSG00000289543.1**	736	1.4	6.5e-05	30,214	-0.1	7.8e-01
**ENSG00000289253.1**	756	1.6	7.1e-05	20,414	-0.4	3.8e-01
**CPNE2-DT**	761	1.4	7.5e-05	32,728	-0.1	8.5e-01
**ENSG00000264666.2**	769	1.3	7.7e-05	22,291	-0.3	4.6e-01
**ENSG00000289119.1**	771	1.4	7.7e-05	5,887	-1.0	5.9e-03
**ENSG00000289067.1**	778	1.4	8.1e-05	4,128	-1.1	1.5e-03
**ENSG00000288942.1**	810	1.5	9.6e-05	13,957	-0.6	1.3e-01
**DYNLL2-DT**	813	1.7	9.8e-05	21,871	-0.4	4.5e-01
**MIR3188**	817	1.5	9.8e-05	22,922	-0.3	4.9e-01
**ENSG00000289267.1**	822	1.8	1.0e-04	40,240	-0.1	9.7e-01
**ENSG00000274092.1**	845	1.6	1.1e-04	26,818	-0.2	6.6e-01
**ENSG00000267882.2**	958	1.6	1.7e-04	31,209	-0.2	8.1e-01
**SMG1-DT**	973	1.5	1.7e-04	12,940	-0.6	1.1e-01
**MAP3K11**	1,001	0.5	1.9e-04	22,995	-0.1	5.0e-01
**ENSG00000289241.1**	1,005	1.4	1.9e-04	40,799	0.0	9.7e-01
**ZFX-AS1**	1,016	1.4	2.0e-04	35,026	-0.1	9.1e-01
**ENSG00000272735.1**	1,025	1.4	2.1e-04	12,399	-0.7	9.4e-02
**ENSG00000273335.1**	1,026	1.5	2.1e-04	25,461	0.3	6.1e-01
**ENSG00000287420.1**	1,037	1.3	2.2e-04	39,718	0.0	9.7e-01
**CETP**	1,061	1.4	2.3e-04	27,248	-0.3	6.8e-01
**ENSG00000279198.1**	1,104	1.2	2.5e-04	25,432	-0.3	6.1e-01
**ENSG00000261519.3**	1,114	1.5	2.6e-04	24,261	-0.3	5.6e-01
**ENSG00000288772.1**	1,122	1.5	2.7e-04	5,381	-0.9	4.1e-03
**ARHGEF2-AS2**	1,144	1.2	2.9e-04	11,463	-0.7	7.2e-02
**ENSG00000288737.1**	1,145	1.5	2.9e-04	17,756	-0.4	2.7e-01
**ENSG00000267212.1**	1,178	1.3	3.3e-04	44,531	0.0	9.9e-01
**ENSG00000279691.1**	1,210	1.5	3.5e-04	27,034	-0.2	6.7e-01
**ENSG00000289330.1**	1,223	1.5	3.6e-04	30,618	-0.2	8.0e-01
**ENSG00000289222.1**	1,235	1.4	3.7e-04	35,135	-0.1	9.1e-01
**OIT3**	1,249	1.7	3.8e-04	20,562	-0.5	3.9e-01
**ENSG00000273141.1**	1,257	1.4	3.9e-04	678	-2.0	9.6e-07
**ENSG00000272195.1**	1,320	1.0	4.5e-04	719	-1.5	1.2e-06
**LINC00115**	1,321	0.9	4.6e-04	2,002	-1.0	7.2e-05
**ENSG00000278546.1**	1,327	1.5	4.6e-04	24,328	-0.3	5.6e-01
**C10ORF95**	1,377	1.4	5.3e-04	24,139	-0.3	5.5e-01
**ENSG00000289518.1**	1,392	1.3	5.6e-04	36,356	-0.1	9.3e-01
**HEXA-AS1**	1,424	1.4	6.1e-04	38,433	-0.1	9.6e-01
**HIGD2B**	1,461	1.3	6.5e-04	30,695	0.2	8.0e-01
**ENSG00000273248.1**	1,462	1.5	6.6e-04	8,365	-0.8	2.3e-02
**ITPKB**	1,507	0.4	7.4e-04	33,932	-0.0	8.9e-01
**ENSG00000262412.1**	1,554	1.3	8.1e-04	17,569	-0.4	2.6e-01
**ENSG00000240790.2**	1,763	1.3	1.2e-03	30,806	-0.2	8.0e-01
**ENSG00000289334.1**	2,102	1.1	2.1e-03	8,873	-0.8	2.9e-02
**MIA2-AS1**	2,224	1.2	2.4e-03	28,956	-0.2	7.4e-01
**OTULIN-DT**	2,300	1.0	2.6e-03	8,470	-0.7	2.4e-02
**ENSG00000272969.1**	2,525	1.3	3.5e-03	32,644	0.1	8.5e-01

	Anti-PM/Scl vs. all			Anti-Mi2 vs. all		
**Gene**	**Pos**	**logFC**	**adj.P.Val**	**Pos**	**logFC**	**adj.P.Val**
**ENSG00000277383.1**	2,662	1.1	4.1e-03	3,356	-1.3	6.6e-04
**ENSG00000272906.1**	2,789	1.0	4.8e-03	12,652	-0.6	1.0e-01
**ENSG00000283341.3**	2,820	0.8	4.9e-03	2,689	-1.1	2.6e-04
**ENSG00000273289.1**	2,852	1.1	5.1e-03	10,618	-0.7	5.5e-02
**ENSG00000279529.1**	3,039	0.8	6.2e-03	2,288	-1.0	1.3e-04
**EXOC3L2**	3,333	0.9	7.8e-03	6,175	-0.9	7.3e-03
**ENSG00000289065.1**	3,437	0.8	8.5e-03	1,028	-1.4	5.5e-06
**ZNF252P-AS1**	4,460	0.9	1.7e-02	17,388	-0.5	2.6e-01
**TNFRSF4**	5,061	0.9	2.4e-02	22,220	-0.4	4.6e-01
**ENSG00000288061.2**	5,690	0.7	3.2e-02	1,745	-1.2	4.2e-05
**ENSG00000277182.1**	5,838	0.8	3.4e-02	30,769	-0.2	8.0e-01
**MHENCR**	15,746	0.3	2.8e-01	4,107	-0.6	1.5e-03
**ENSG00000280152.1**	16,487	0.5	3.0e-01	718	-1.7	1.1e-06
**ENSG00000276570.1**	39,493	0.1	8.9e-01	3,518	-1.0	8.0e-04

Supplementary Table 3. Differential expression of anti-PM/Scl-specific genes (PMID 38902010) in muscle biopsies from anti-PM/Scl-positive patients, relative to all other samples, in an independent external validation cohort. Pos, position in the complete differential expression table; logFC, log_2 fold change; adj.P.Val, Benjamini-Hochberg-adjusted P value.

Gene	Pos	logFC	adj.P.Val
**ENSG00000265100**	1	2.9	1.8e-10
**ENSG00000268403**	6	2.6	1.1e-08
**ENSG00000289296**	7	2.4	1.2e-08
**ENSG00000260369**	8	3.4	1.2e-08
**ENSG00000272735**	13	2.7	2.2e-08
**ENSG00000239705**	17	4.0	2.5e-08
**ENSG00000289499**	20	2.3	3.8e-08
**ENSG00000289637**	22	3.3	3.8e-08
**ENSG00000287821**	29	3.6	4.0e-08
**CAPN10-DT**	30	1.6	5.8e-08
**BMP2K-DT**	33	3.2	6.8e-08
**ENSG00000289030**	37	2.4	9.6e-08
**ENSG00000288804**	41	3.2	1.9e-07
**ENSG00000279212**	52	3.0	2.8e-07
**ENSG00000287584**	53	1.9	3.1e-07
**ENSG00000286408**	59	2.5	3.5e-07
**ENSG00000273210**	60	2.7	3.5e-07
**MIR5188**	61	4.2	3.5e-07
**ENSG00000287547**	71	3.3	4.7e-07
**TTC32-DT**	84	2.9	7.4e-07
**DDX39B-AS1**	100	2.8	1.0e-06
**HIGD2B**	105	3.5	1.1e-06
**TRIM51BP**	108	4.7	1.1e-06
**ENSG00000289119**	116	2.5	1.3e-06
**ENSG00000288996**	121	3.2	1.5e-06
**ENSG00000289115**	122	3.4	1.5e-06
**ENSG00000288900**	147	3.0	2.3e-06
**ENSG00000286409**	149	2.7	2.4e-06
**ENSG00000273141**	158	2.3	2.8e-06
**ENSG00000289253**	159	2.1	2.8e-06
**TIMMDC1-DT**	169	2.9	3.5e-06
**ENSG00000261242**	171	2.5	3.7e-06
**ENSG00000273338**	182	4.6	5.0e-06
**ENSG00000272967**	186	2.9	5.3e-06
**ENSG00000289303**	187	1.7	5.3e-06
**ENSG00000255647**	205	2.0	7.5e-06
**ENSG00000272768**	223	2.1	9.8e-06
**ENSG00000269399**	244	1.8	1.3e-05
**ENSG00000276524**	256	2.0	1.5e-05
**ENSG00000240790**	258	2.3	1.5e-05
**ENSG00000278017**	267	1.8	1.7e-05
**ENSG00000284484**	283	4.2	2.0e-05
**ENSG00000286113**	296	3.0	2.2e-05
**ENSG00000274292**	297	1.6	2.2e-05
**ENSG00000234432**	300	1.6	2.4e-05
**AFF4-DT**	301	2.3	2.4e-05
**ENSG00000288872**	316	3.0	3.0e-05
**ENSG00000288865**	322	2.5	3.2e-05
**ENSG00000288751**	323	1.7	3.2e-05
**THOC1-DT**	324	1.4	3.2e-05
**UAP1-DT**	331	2.0	3.3e-05

Gene	Pos	logFC	adj.P.Val
**SLC38A2-AS1**	349	2.5	3.8e-05
**ENSG00000288988**	357	1.8	3.9e-05
**ENSG00000277383**	364	1.8	4.2e-05
**ENSG00000289229**	369	2.5	4.4e-05
**ENSG00000286444**	384	3.0	5.1e-05
**LINC02776**	388	2.7	5.4e-05
**ENSG00000272906**	397	1.6	6.3e-05
**ENSG00000287070**	404	1.6	6.6e-05
**ENSG00000287697**	423	1.3	9.2e-05
**ENSG00000289412**	435	2.1	1.1e-04
**ENSG00000274213**	444	2.4	1.1e-04
**ENSG00000273335**	454	2.7	1.3e-04
**ENSG00000289230**	457	2.4	1.3e-04
**MIR378D2HG**	458	2.3	1.3e-04
**PRAMEF13**	465	2.4	1.4e-04
**ENSG00000289341**	474	2.9	1.4e-04
**LINC01424**	485	1.6	1.7e-04
**ENSG00000286881**	489	2.2	1.7e-04
**ENSG00000288744**	490	1.2	1.7e-04
**ENSG00000289200**	501	3.0	1.9e-04
**ENSG00000289202**	536	2.8	2.5e-04
**ENSG00000272195**	546	1.4	2.7e-04
**RN7SL521P**	549	2.1	2.8e-04
**LINC00677**	550	2.3	2.9e-04
**ENSG00000245651**	556	2.0	3.0e-04
**ENSG00000225945**	558	1.9	3.0e-04
**CPEB2-DT**	570	1.5	3.2e-04
**ARHGEF2-AS2**	571	1.3	3.3e-04
**ENSG00000289031**	622	1.1	4.7e-04
**SMG1-DT**	624	1.4	4.8e-04
**ENSG00000288879**	632	2.7	5.0e-04
**ENSG00000289317**	645	2.1	5.3e-04
**ENSG00000272426**	658	2.0	5.7e-04
**ENSG00000289379**	664	2.0	5.9e-04
**ENSG00000289152**	687	2.1	7.5e-04
**ENSG00000278743**	692	1.4	7.9e-04
**ENSG00000287979**	702	2.8	8.5e-04
**ENSG00000279140**	717	1.4	9.5e-04
**ENSG00000272969**	729	2.3	1.0e-03
**DYNLL2-DT**	734	2.3	1.0e-03
**ENSG00000287654**	746	2.1	1.1e-03
**ENSG00000289334**	759	1.7	1.2e-03
**ENSG00000275709**	763	1.7	1.3e-03
**ENSG00000276744**	764	1.6	1.3e-03
**ENSG00000289301**	768	2.4	1.4e-03
**ENSG00000289055**	772	1.8	1.4e-03
**ENSG00000264666**	790	1.7	1.6e-03
**ENSG00000289257**	810	2.2	1.9e-03
**ENSG00000276216**	816	2.1	1.9e-03
**ENSG00000289221**	822	2.6	2.0e-03
**ENSG00000261334**	829	1.8	2.1e-03
**ENSG00000289065**	831	1.6	2.2e-03
**ENSG00000277020**	842	1.6	2.3e-03
**ZFX-AS1**	870	2.1	2.6e-03
**ENSG00000267882**	911	1.7	2.9e-03
**ENSG00000288746**	935	1.3	3.4e-03
**ENSG00000288919**	948	1.2	3.6e-03
**LINC01126**	979	0.8	4.1e-03

Gene	Pos	logFC	adj.P.Val
**ENSG00000289154**	1,006	0.8	4.7e-03
**ENSG00000289267**	1,022	2.5	5.0e-03
**ENSG00000286577**	1,025	1.6	5.1e-03
**ENSG00000289288**	1,047	1.7	5.8e-03
**ENSG00000288835**	1,067	1.6	6.3e-03
**ENSG00000288955**	1,086	1.8	6.9e-03
**ENSG00000260651**	1,113	1.9	8.1e-03
**ENSG00000289457**	1,120	1.0	8.2e-03
**SMG7-AS1**	1,139	1.3	9.2e-03
**CPNE2-DT**	1,150	2.0	9.8e-03
**ENSG00000286482**	1,153	0.8	9.9e-03
**ENSG00000289550**	1,157	2.6	1.0e-02
**TRIM8-DT**	1,198	1.3	1.2e-02
**ENSG00000288942**	1,255	0.9	1.5e-02
**MSRA-DT**	1,267	1.0	1.6e-02
**ENSG00000268670**	1,271	0.9	1.6e-02
**ENSG00000224505**	1,310	0.7	1.8e-02
**ENSG00000288927**	1,347	1.0	2.1e-02
**CAGE1**	1,350	2.1	2.1e-02
**ENSG00000274751**	1,392	1.4	2.5e-02
**HEXA-AS1**	1,430	1.2	2.8e-02
**ENSG00000279259**	1,432	1.8	2.9e-02
**ENSG00000289222**	1,444	2.0	3.0e-02
**ENSG00000289551**	1,463	0.8	3.0e-02
**MIA2-AS1**	1,469	1.2	3.1e-02
**ENSG00000288842**	1,529	1.6	3.6e-02
**ENSG00000273064**	1,542	0.9	3.7e-02
**ENSG00000289506**	1,564	1.2	4.0e-02
**ENSG00000289103**	1,610	0.7	4.4e-02
**ENSG00000241666**	1,621	1.0	4.5e-02
**ATXN7L3-AS1**	1,652	0.6	4.9e-02
**OTULIN-DT**	1,678	1.0	5.2e-02
**SDR42E2**	1,681	1.3	5.2e-02
**ENSG00000259135**	1,687	1.3	5.3e-02
**ENSG00000278002**	1,716	0.8	5.6e-02
**ENSG00000264739**	1,844	1.4	7.4e-02
**CLMAT3**	1,864	1.5	7.6e-02
**ENSG00000289182**	1,873	0.9	7.8e-02
**ENSG00000279491**	1,929	1.2	8.8e-02
**KLF2-DT**	1,940	0.9	8.9e-02
**ENSG00000288061**	1,986	0.7	9.4e-02
**MIR23AHG**	2,142	0.9	1.1e-01
**ENSG00000279529**	2,154	0.4	1.1e-01
**ENSG00000289067**	2,206	0.9	1.2e-01
**ITPKB**	2,216	-0.5	1.2e-01
**ENSG00000288939**	2,232	1.2	1.2e-01
**ENSG00000270012**	2,236	0.5	1.2e-01
**ENSG00000289543**	2,363	1.7	1.4e-01
**ENSG00000257258**	2,575	1.5	1.7e-01
**RENO1**	2,655	0.4	1.9e-01
**ANKH-DT**	2,705	1.4	1.9e-01
**ENSG00000267212**	2,771	0.8	2.0e-01
**MHENCR**	2,805	0.6	2.0e-01
**ENSG00000279691**	3,011	0.7	2.3e-01
**LINC01089**	3,242	0.5	2.6e-01
**TERC**	3,387	0.6	2.7e-01
**ENSG00000289330**	3,641	1.3	3.0e-01
**ENSG00000277182**	3,893	0.8	3.2e-01

Gene	Pos	logFC	adj.P.Val
**ENSG00000283959**	3,907	0.8	3.2e-01
**ENSG00000289059**	3,988	0.7	3.3e-01
**ENSG00000262412**	4,355	0.7	3.6e-01
**ENSG00000289547**	4,514	0.8	3.7e-01
**SPACA6P-AS**	4,803	0.9	3.9e-01
**COL18A1**	5,251	0.6	4.2e-01
**ENSG00000288813**	5,262	1.3	4.2e-01
**EGFL7**	5,757	0.4	4.5e-01
**ENSG00000289159**	5,878	1.1	4.6e-01
**ZNF252P-AS1**	6,237	0.4	4.8e-01
**ENSG00000288929**	7,175	0.6	5.2e-01
**ENSG00000230695**	7,235	1.0	5.3e-01
**EXOC3L2**	7,589	0.7	5.4e-01
**ENSG00000274251**	9,262	0.3	6.1e-01
**MIRLET7BHG**	9,396	0.2	6.1e-01
**ENSG00000288866**	9,854	0.4	6.3e-01
**LINC00896**	9,985	0.5	6.3e-01
**ENSG00000289177**	10,001	0.2	6.3e-01
**ENSG00000280152**	10,935	0.2	6.6e-01
**ENSG00000278932**	11,098	0.4	6.6e-01
**TNFRSF4**	15,102	0.5	7.5e-01
**TNFAIP8L1**	17,598	0.2	8.0e-01
**ENSG00000278546**	18,146	0.4	8.1e-01
**ENSG00000289235**	19,171	0.4	8.2e-01
**OIT3**	19,402	0.5	8.3e-01
**ENSG00000274737**	20,140	0.2	8.4e-01
**MIR1915HG**	21,234	0.3	8.6e-01
**ENSG00000287420**	21,675	0.4	8.6e-01
**FSCN1**	22,834	0.3	8.8e-01
**ENSG00000274092**	23,199	0.3	8.8e-01
**PRND**	23,378	0.7	8.9e-01
**ENSG00000282936**	25,015	0.2	9.1e-01
**ENSG00000289044**	25,415	0.1	9.1e-01
**ENSG00000280035**	26,813	-0.1	9.3e-01
**MAP3K11**	26,947	0.1	9.3e-01
**ENSG00000289518**	26,948	-0.2	9.3e-01
**ENSG00000273248**	27,246	0.1	9.3e-01
**ENSG00000276570**	28,986	-0.1	9.5e-01
**ENSG00000253838**	29,287	-0.2	9.5e-01
**LINC00115**	33,981	0.0	1.0e+00
**RABEP2**	34,012	-0.0	1.0e+00
**CETP**	34,071	-0.0	1.0e+00

Supplementary Table 4. Association between a previously defined set of anti-Mi2- and anti-PM/Scl-specific genes (PMID 38902010) and genes differentially expressed (q-value < 0.001) at 24h and 72h following electroporation of purified immunoglobulins into primary muscle cell cultures, relative to other electroporated samples. Anti-Mi2-specific genes are preferentially enriched at 24h post-electroporation, whereas anti-PM/Scl-specific genes are preferentially enriched at 72h post-electroporation. IBM, inclusion body myositis; ACA, anti-centromere autoantibodies.

24h after electroporation

Group	Mi2	Not Mi2	p-value
Control	0% (0)	0% (0)	1
Mi2	66% (75)	2% (1196)	<2e-16
PM/Scl	1% (1)	2% (937)	1
NXP2	0% (0)	0% (0)	1
TIF1	0% (0)	0% (2)	1
MDA5	0% (0)	0% (46)	1
Jo1	0% (0)	0% (0)	1
HMGCR	0% (0)	0% (0)	1
SRP	0% (0)	0% (3)	1
IBM	0% (0)	0% (2)	1
Ku	0% (0)	0% (10)	1
Scl70	1% (1)	1% (572)	1
ACA	0% (0)	0% (11)	1

Group	PM/Scl	Not PM/Scl	p-value
Control	0% (0)	0% (0)	1
Mi2	1% (2)	2% (1269)	0.25
PM/Scl	69% (163)	1% (775)	<2e-16
NXP2	0% (0)	0% (0)	1
TIF1	0% (0)	0% (2)	1
MDA5	0% (0)	0% (46)	1
Jo1	0% (0)	0% (0)	1
HMGCR	0% (0)	0% (0)	1
SRP	0% (0)	0% (3)	1
IBM	0% (0)	0% (2)	1
Ku	0% (0)	0% (10)	1
Scl70	0% (0)	1% (573)	0.18
ACA	0% (0)	0% (11)	1

72h after electroporation

Group	Mi2	Not Mi2	p-value
Control	0% (0)	0% (0)	1
Mi2	49% (55)	1% (435)	<2e-16
PM/Scl	3% (3)	3% (1903)	1
NXP2	0% (0)	0% (0)	1
TIF1	0% (0)	0% (12)	1
MDA5	0% (0)	0% (0)	1
Jo1	0% (0)	0% (0)	1
HMGCR	0% (0)	0% (1)	1
SRP	0% (0)	0% (0)	1
IBM	0% (0)	0% (0)	1
Ku	0% (0)	0% (3)	1
Scl70	0% (0)	0% (87)	1
ACA	0% (0)	0% (63)	1

Group	PM/Scl	Not PM/Scl	p-value
Control	0% (0)	0% (0)	1
Mi2	2% (4)	1% (486)	0.12
PM/Scl	86% (202)	3% (1704)	<2e-16
NXP2	0% (0)	0% (0)	1
TIF1	0% (0)	0% (12)	1
MDA5	0% (0)	0% (0)	1
Jo1	0% (0)	0% (0)	1
HMGCR	0% (0)	0% (1)	1
SRP	0% (0)	0% (0)	1
IBM	0% (0)	0% (0)	1
Ku	0% (0)	0% (3)	1
Scl70	0% (0)	0% (87)	1
ACA	0% (0)	0% (63)	1

Supplementary Table 5. Differential expression of anti-Mi2-specific genes (PMID 38902010) at 24h and 72h following electroporation of purified immunoglobulins from anti-Mi2-positive patients into primary muscle cell cultures, relative to other electroporated conditions. Pos, position in the complete differential expression table; logFC, log_2 fold change; adj.P.Val, Benjamini-Hochberg-adjusted P value.

	Mi2-specific genes at 24h			Mi2-specific genes at 72h		
**Gene**	**Pos**	**logFC**	**adj.P.Val**	**Pos**	**logFC**	**adj.P.Val**
**TEX19**	2	4.5	3.6e-24	317	2.5	4.3e-05
**TMEM151A**	3	3.5	3.6e-24	278	1.6	1.4e-05
**CAMKV**	4	6.1	6.0e-23	324	2.6	5.5e-05
**COX6B2**	9	4.2	4.4e-22	32	3.3	1.9e-15
**PLPPR3**	10	3.4	8.8e-22	262	1.5	6.8e-06
**PPP1R1B**	12	5.8	1.7e-21	53	3.6	4.0e-13
**BAIAP3**	16	3.4	7.2e-21	70	1.8	1.4e-11
**MSI1**	17	3.0	7.2e-21	259	1.3	5.9e-06
**TMEM151B**	25	4.0	1.9e-19	207	2.6	5.0e-07
**LINC00624**	27	4.8	2.2e-19	55	3.5	6.7e-13
**KIF1A**	32	3.5	6.7e-19	54	2.4	4.1e-13
**RAP1GAP**	34	2.9	1.7e-18	1,688	0.7	1.5e-01
**P2RX2**	39	4.5	8.6e-18	108	3.6	5.9e-10
**CACNA1G**	43	3.0	1.8e-17	89	2.4	8.1e-11
**JPH3**	44	3.4	1.8e-17	200	2.5	3.2e-07
**PALM3**	47	3.7	2.2e-17	335	1.5	7.1e-05
**CAPN12**	55	3.0	5.6e-17	183	1.6	1.5e-07
**DLGAP3**	57	3.6	8.2e-17	161	2.5	4.2e-08
**KLC3**	62	2.9	1.3e-16	468	1.5	7.1e-04
**LGI3**	67	3.4	2.0e-16	43	3.1	2.1e-14
**FLRT1**	70	3.0	3.0e-16	4,037	0.9	4.4e-01
**BANCR**	74	4.1	8.9e-16	123	2.7	2.5e-09
**ANKRD20A19P**	80	3.7	1.8e-15	99	3.4	2.2e-10
**IGLON5**	82	3.6	2.4e-15	62	3.5	2.7e-12
**CAMSAP3**	86	3.4	4.7e-15	189	2.6	2.2e-07
**ABCG4**	95	2.9	1.1e-14	598	1.1	3.7e-03
**TMEM145**	96	2.6	1.1e-14	218	1.7	8.0e-07
**CADM4**	97	1.8	1.3e-14	143	1.4	1.3e-08
**CHGA**	108	3.9	7.2e-14	128	2.8	4.0e-09
**COL2A1**	109	2.9	9.2e-14	211	1.9	6.1e-07
**SPIB**	120	3.0	1.9e-13	164	2.1	4.6e-08
**EFNA3**	126	1.9	2.9e-13	567	0.9	2.6e-03
**UTF1**	127	4.0	3.8e-13	273	2.5	1.1e-05
**CLDN6**	138	2.4	7.8e-13	254	1.4	3.7e-06
**SLC29A4**	156	2.0	3.5e-12	432	0.9	4.9e-04
**PRR35**	173	3.5	7.3e-12	472	1.7	7.7e-04
**HPCA**	179	2.6	9.3e-12	210	1.9	6.0e-07
**CASKIN1**	180	1.7	1.0e-11	371	0.8	1.8e-04
**YBX2**	181	2.6	1.1e-11	599	1.3	3.8e-03
**NKAIN4**	187	3.0	1.2e-11	479	1.8	9.2e-04
**DACT3**	200	2.1	2.8e-11	167	1.1	5.4e-08
**KCNJ4**	201	2.6	2.9e-11	229	1.8	1.8e-06
**SHISA7**	229	4.3	1.1e-10	180	3.3	1.1e-07
**SEMA4G**	237	1.8	1.7e-10	808	0.4	2.0e-02
**WFDC2**	239	3.7	1.9e-10	50	4.2	3.3e-13
**CTSV**	257	1.4	4.7e-10	348	1.2	8.5e-05
**CPNE6**	258	3.3	4.9e-10	45	2.7	8.4e-14
**CHRM4**	273	2.2	8.0e-10	893	1.3	2.9e-02
**ECE2**	284	4.9	1.4e-09	119	4.1	1.7e-09
**ZNF467**	290	3.0	1.6e-09	746	1.8	1.2e-02

	Mi2-specific genes at 24h			Mi2-specific genes at 72h		
**Gene**	**Pos**	**logFC**	**adj.P.Val**	**Pos**	**logFC**	**adj.P.Val**
**TH**	299	2.3	2.1e-09	430	1.5	4.7e-04
**OVOL1**	371	2.9	1.4e-08	170	2.6	6.1e-08
**B4GALNT4**	388	1.9	2.3e-08	483	1.6	9.4e-04
**SCRT1**	456	2.4	1.3e-07	744	1.4	1.2e-02
**GCGR**	483	2.5	2.2e-07	391	2.4	2.3e-04
**ENSG00000289332.1**	511	2.7	4.0e-07	185	3.0	1.6e-07
**ENSG00000169093.16**	530	0.8	6.7e-07	454	0.6	6.3e-04
**KCNQ2**	539	2.8	7.5e-07	303	2.8	2.5e-05
**STAC2**	545	1.5	7.9e-07	2,934	0.6	3.2e-01
**DHRS2**	574	1.9	1.5e-06	413	2.1	3.6e-04
**CRB3**	605	2.7	2.7e-06	805	1.6	1.9e-02
**FBXL16**	662	1.7	6.8e-06	588	0.9	3.3e-03
**CACNA1I**	728	2.3	1.5e-05	761	1.7	1.4e-02
**ENSG00000286311.1**	732	1.5	1.6e-05	14,714	0.3	8.4e-01
**SMIM24**	783	1.8	2.6e-05	384	2.6	2.0e-04
**FAM171A2**	853	1.2	5.1e-05	6,716	0.5	6.2e-01
**GRIN3B**	964	1.6	1.4e-04	3,901	0.8	4.3e-01
**RAB26**	968	1.9	1.5e-04	310	1.1	3.3e-05
**RAB3B**	986	0.9	1.7e-04	17,260	-0.1	8.9e-01
**HCN2**	1,025	0.6	2.2e-04	1,536	0.5	1.2e-01
**ARHGDIG**	1,036	1.4	2.5e-04	529	1.4	1.7e-03
**ESPN**	1,065	1.1	3.2e-04	3,736	0.7	4.1e-01
**SOX15**	1,175	1.1	6.3e-04	2,241	0.5	2.3e-01
**TNNI3**	1,176	1.6	6.3e-04	1,134	1.1	6.2e-02
**IFITM5**	1,209	2.4	7.4e-04	707	1.6	9.8e-03
**PRKAR1B**	1,284	0.7	1.1e-03	2,082	0.4	2.1e-01
**GRIN2D**	1,287	1.2	1.1e-03	6,809	0.5	6.3e-01
**LKAAEAR1**	1,291	2.1	1.1e-03	1,168	1.5	6.8e-02
**IL11**	1,424	1.3	2.3e-03	23,689	-0.1	9.8e-01
**KREMEN2**	1,643	1.2	5.3e-03	11,034	0.5	7.6e-01
**PDIA2**	1,693	0.8	6.3e-03	398	1.8	2.6e-04
**ENTPD8**	1,736	1.9	7.1e-03	17,744	0.2	9.0e-01
**ENSG00000267892.1**	1,874	1.2	1.0e-02	1,853	0.6	1.8e-01
**PRKCG**	2,093	0.6	1.6e-02	778	1.0	1.6e-02
**CBARP**	2,747	0.5	4.2e-02	5,638	0.2	5.6e-01
**AQP5**	2,816	1.2	4.6e-02	650	1.1	5.9e-03
**ENSG00000260293.2**	2,842	1.0	4.7e-02	6,219	0.5	6.0e-01
**SSU72P8**	3,168	0.7	6.7e-02	81	2.7	3.1e-11
**COL26A1**	3,545	1.0	8.8e-02	2,092	1.3	2.1e-01
**VWA5B2**	5,107	0.9	2.1e-01	1,849	0.9	1.8e-01
**MADCAM1**	7,365	0.5	3.9e-01	571	1.3	2.6e-03
**ENSG00000275437.1**	8,813	0.3	4.8e-01	22,714	0.1	9.7e-01
**GJD2**	9,051	-0.6	4.9e-01	509	1.3	1.3e-03
**ENSG00000218416.4**	9,411	0.5	5.1e-01	11,984	0.5	7.8e-01
**KBTBD11-AS1**	10,062	0.6	5.5e-01	17,934	-0.3	9.0e-01
**ALPG**	20,050	0.2	9.0e-01	268	3.4	7.6e-06
**ENSG00000223561.7**	20,277	0.2	9.0e-01	6,002	0.7	5.8e-01
**RAC3**	23,126	0.0	9.6e-01	7,238	0.3	6.4e-01
**CT69**	23,950	-0.1	9.7e-01	13,098	0.4	8.1e-01
**FAM131C**	24,144	0.0	9.8e-01	13,940	-0.2	8.3e-01

Supplementary Table 6. Differential expression of anti-PM/Scl-specific genes (PMID 38902010) at 72h and 24h following electroporation of purified immunoglobulins from anti-PM/Scl-positive patients into primary muscle cell cultures, relative to other electroporated conditions. Pos, position in the complete differential expression table; logFC, log_2 fold change; adj.P.Val, Benjamini-Hochberg-adjusted P value.

	PM/Scl-specific genes at 72h			PM/Scl-specific genes at 24h		
**Gene**	**Pos**	**logFC**	**adj.P.Val**	**Pos**	**logFC**	**adj.P.Val**
**ENSG00000226380.9**	2	5.0	3.4e-25	128	2.6	2.4e-07
**ENSG00000288865.1**	3	5.2	5.0e-21	549	2.8	7.9e-05
**ENSG00000274292.1**	6	4.7	8.1e-21	52	2.6	1.9e-08
**ENSG00000288751.1**	8	6.1	5.2e-20	22	4.2	2.0e-09
**ENSG00000289543.1**	9	6.3	7.9e-20	33	4.6	9.6e-09
**MIRLET7BHG**	11	2.3	2.1e-19	362	0.8	1.4e-05
**ENSG00000272426.1**	12	4.8	1.5e-18	176	3.4	7.7e-07
**ENSG00000288955.1**	14	5.9	2.5e-18	138	4.3	3.0e-07
**ENSG00000288900.1**	20	6.4	1.6e-17	62	4.4	2.5e-08
**ENSG00000289412.1**	21	3.1	4.0e-17	212	2.5	1.6e-06
**ENSG00000234432.4**	23	4.1	4.1e-17	26	3.3	5.5e-09
**ENSG00000239705.2**	24	6.3	4.6e-17	4	4.3	1.3e-10
**ENSG00000287547.1**	26	5.0	1.2e-16	35	3.5	1.1e-08
**ENSG00000278017.1**	28	5.1	1.4e-16	783	2.5	3.5e-04
**ENSG00000289030.1**	30	6.0	1.7e-16	17	4.7	1.0e-09
**ENSG00000289059.1**	36	4.4	3.3e-16	47	3.3	1.6e-08
**ENSG00000276216.1**	38	6.3	4.7e-16	32	4.2	9.6e-09
**ENSG00000273338.1**	39	4.6	5.2e-16	175	3.6	7.7e-07
**ENSG00000230695.2**	40	4.6	5.5e-16	100	3.5	8.5e-08
**ENSG00000261334.1**	41	6.4	5.8e-16	8	4.5	3.0e-10
**ENSG00000273210.1**	43	4.9	5.8e-16	13	3.5	7.5e-10
**ENSG00000289257.1**	44	4.6	7.0e-16	92	3.3	8.1e-08
**ENSG00000289200.1**	47	5.2	7.5e-16	434	3.1	2.3e-05
**ENSG00000289044.1**	48	5.2	8.2e-16	121	2.7	1.7e-07
**ENSG00000289457.1**	56	4.9	1.4e-15	658	3.0	1.6e-04
**TTC32-DT**	58	5.1	1.5e-15	126	3.4	2.2e-07
**ENSG00000278002.1**	60	3.1	1.6e-15	74	2.4	4.5e-08
**ENSG00000272967.1**	62	4.2	1.7e-15	604	2.5	1.1e-04
**ATXN7L3-AS1**	67	4.3	2.9e-15	929	2.4	9.1e-04
**ENSG00000265100.1**	74	4.9	6.1e-15	75	3.5	4.5e-08
**ENSG00000287979.1**	76	5.9	6.8e-15	990	2.5	1.3e-03
**ENSG00000289288.1**	78	3.8	7.0e-15	1,569	1.9	1.8e-02
**ENSG00000288835.1**	79	4.4	7.2e-15	10	3.8	5.3e-10
**ENSG00000289550.1**	80	3.2	9.0e-15	285	2.5	5.2e-06
**ENSG00000289341.1**	81	4.8	1.1e-14	715	2.5	2.4e-04
**ENSG00000272735.1**	82	5.4	1.2e-14	116	3.5	1.1e-07
**ENSG00000279212.1**	83	5.2	1.6e-14	258	3.7	4.2e-06
**CLMAT3**	85	4.7	2.2e-14	55	3.4	2.1e-08
**ENSG00000254028.1**	89	4.2	3.1e-14	535	2.4	7.0e-05
**ENSG00000289103.1**	93	4.7	3.4e-14	229	3.5	2.3e-06
**ENSG00000286409.2**	95	4.5	3.9e-14	27	3.7	6.3e-09
**ENSG00000272195.1**	96	3.9	4.1e-14	85	2.9	6.1e-08
**ENSG00000260369.2**	97	5.5	4.1e-14	34	4.3	1.0e-08
**ENSG00000225945.1**	98	4.3	4.6e-14	683	2.5	2.0e-04
**AFF4-DT**	102	4.5	5.8e-14	718	2.5	2.5e-04
**ENSG00000287584.1**	104	4.4	5.9e-14	251	3.3	3.5e-06
**ENSG00000289221.1**	108	4.5	7.3e-14	143	3.8	3.2e-07
**HEXA-AS1**	109	3.1	7.3e-14	2	3.2	1.0e-10
**ENSG00000288929.1**	111	4.8	7.4e-14	159	3.4	4.6e-07
**ENSG00000274751.1**	115	2.8	8.1e-14	820	1.6	4.5e-04
**ENSG00000289267.1**	116	4.5	8.3e-14	418	3.1	2.1e-05
**ENSG00000279491.1**	121	3.9	1.1e-13	179	2.9	8.2e-07
**MIR378D2HG**	122	5.0	1.2e-13	202	3.5	1.4e-06
**LINC01424**	124	3.7	1.4e-13	95	2.5	8.4e-08
**ENSG00000289115.1**	125	5.1	1.6e-13	480	2.9	4.1e-05
**ENSG00000289119.1**	127	2.7	1.7e-13	67	2.1	3.5e-08

	PM/Scl-specific genes at 72h			PM/Scl-specific genes at 24h		
**Gene**	**Pos**	**logFC**	**adj.P.Val**	**Pos**	**logFC**	**adj.P.Val**
**ENSG00000288942.1**	128	3.4	1.7e-13	740	2.2	2.8e-04
**ENSG00000224505.3**	129	3.2	1.7e-13	97	2.6	8.5e-08
**ENSG00000289330.1**	130	3.7	1.7e-13	1,196	2.0	4.0e-03
**ENSG00000289055.1**	136	4.0	2.3e-13	1,035	2.2	1.6e-03
**ENSG00000288943.1**	138	5.2	2.3e-13	288	3.2	5.5e-06
**ENSG00000277020.4**	140	3.9	2.4e-13	336	2.9	1.0e-05
**ENSG00000287697.1**	145	3.9	2.6e-13	135	2.4	2.9e-07
**ENSG00000286881.1**	146	4.2	2.7e-13	149	3.1	3.7e-07
**TRIM8-DT**	147	3.1	3.4e-13	201	2.2	1.3e-06
**ENSG00000274092.1**	149	4.1	3.9e-13	29	3.3	7.4e-09
**SLC38A2-AS1**	151	4.5	4.1e-13	152	3.4	4.1e-07
**SPACA6P-AS**	153	3.2	5.4e-13	331	2.0	9.8e-06
**BMP2K-DT**	154	3.9	5.5e-13	61	2.9	2.5e-08
**ENSG00000245651.3**	157	4.8	6.3e-13	537	2.6	7.3e-05
**ENSG00000286444.1**	163	4.7	6.8e-13	368	2.8	1.5e-05
**ENSG00000289065.1**	165	3.4	6.9e-13	111	2.2	1.1e-07
**ENSG00000289626.1**	169	2.6	7.8e-13	478	1.5	4.0e-05
**THOC1-DT**	174	3.2	9.9e-13	270	2.4	4.8e-06
**ENSG00000288872.1**	175	4.9	1.0e-12	190	3.3	1.1e-06
**ENSG00000268403.2**	178	3.4	1.2e-12	132	2.3	2.6e-07
**ENSG00000289506.1**	184	3.9	1.6e-12	88	3.1	6.7e-08
**ENSG00000287821.1**	185	4.6	1.6e-12	241	2.9	2.8e-06
**ENSG00000272719.1**	192	4.1	2.3e-12	187	3.3	1.0e-06
**ENSG00000284602.1**	195	2.4	2.4e-12	39	2.0	1.1e-08
**ENSG00000240790.2**	197	4.4	2.6e-12	697	2.5	2.1e-04
**ENSG00000259135.1**	200	4.2	2.7e-12	14,630	0.7	7.3e-01
**ENSG00000289142.1**	203	4.1	3.2e-12	257	2.5	4.1e-06
**SMG7-AS1**	215	2.9	4.4e-12	284	2.4	5.2e-06
**ENSG00000286408.1**	225	3.3	5.5e-12	155	2.2	4.3e-07
**MIA2-AS1**	229	2.6	5.7e-12	807	1.6	3.9e-04
**ENSG00000289637.1**	234	4.2	6.2e-12	1,085	2.1	2.1e-03
**ENSG00000288804.1**	241	4.5	7.4e-12	38	3.4	1.1e-08
**ENSG00000289301.1**	245	4.4	8.4e-12	620	2.8	1.2e-04
**ENSG00000289317.1**	248	4.4	8.7e-12	701	2.8	2.1e-04
**ENSG00000273064.1**	255	2.5	1.0e-11	86	2.3	6.2e-08
**ENSG00000264666.2**	258	2.8	1.1e-11	145	2.1	3.3e-07
**ENSG00000286577.1**	259	4.0	1.1e-11	539	2.5	7.3e-05
**ENSG00000289296.1**	268	3.9	1.4e-11	139	2.9	3.1e-07
**ENSG00000255647.3**	270	4.0	1.4e-11	106	3.3	9.8e-08
**ENSG00000288988.1**	272	3.2	1.6e-11	188	2.2	1.0e-06
**ENSG00000288996.1**	275	4.2	1.6e-11	865	2.3	6.9e-04
**ENSG00000277182.1**	276	2.0	1.7e-11	127	1.7	2.2e-07
**ENSG00000288866.1**	282	4.0	1.8e-11	240	3.1	2.8e-06
**ENSG00000278743.1**	289	3.4	2.2e-11	580	2.3	9.4e-05
**MHENCR**	297	1.3	2.8e-11	460	0.9	3.2e-05
**ENSG00000283341.3**	299	1.9	2.9e-11	696	1.0	2.1e-04
**ENSG00000288061.2**	302	2.2	3.2e-11	338	1.7	1.1e-05
**ENSG00000289303.1**	304	3.4	3.5e-11	428	2.5	2.2e-05
**ENSG00000289499.1**	321	4.3	5.1e-11	136	3.1	2.9e-07
**ENSG00000288927.1**	328	3.5	5.3e-11	198	3.4	1.3e-06
**ENSG00000286482.1**	329	2.0	5.3e-11	405	2.0	1.9e-05
**ENSG00000289518.1**	332	3.0	5.7e-11	530	2.3	6.8e-05
**ZNF252P-AS1**	333	2.0	5.9e-11	772	1.7	3.3e-04
**ENSG00000272953.1**	334	2.9	6.3e-11	72	2.5	4.0e-08
**ENSG00000288896.1**	338	3.6	6.8e-11	158	2.8	4.6e-07
**ZFX-AS1**	339	4.1	6.8e-11	928	2.1	9.1e-04
**ENSG00000261519.3**	341	3.2	7.5e-11	1,721	1.5	2.7e-02
**ENSG00000261242.1**	343	3.0	7.7e-11	688	2.1	2.0e-04
**ENSG00000276524.1**	346	3.4	8.3e-11	383	2.6	1.7e-05
**ENSG00000278932.5**	348	1.9	8.8e-11	45	1.3	1.5e-08
**ENSG00000267882.2**	355	4.0	1.0e-10	162	3.0	5.4e-07
**MSRA-DT**	365	3.3	1.2e-10	246	3.2	3.1e-06
**ENSG00000289031.1**	379	3.4	1.5e-10	442	2.8	2.5e-05
**ENSG00000269399.2**	380	2.0	1.5e-10	365	1.3	1.4e-05
**ARHGEF2-AS2**	383	2.2	1.6e-10	134	1.6	2.8e-07

	PM/Scl-specific genes at 72h			PM/Scl-specific genes at 24h		
**Gene**	**Pos**	**logFC**	**adj.P.Val**	**Pos**	**logFC**	**adj.P.Val**
**ENSG00000289222.1**	389	3.3	1.8e-10	160	2.9	4.7e-07
**DDX39B-AS1**	399	3.8	2.3e-10	204	3.0	1.4e-06
**CAGE1**	406	3.2	2.6e-10	1,868	1.5	4.0e-02
**ENSG00000289154.1**	408	2.0	2.6e-10	350	1.4	1.2e-05
**TERC**	411	4.8	2.6e-10	977	2.1	1.2e-03
**CAPN10-DT**	422	1.8	3.2e-10	231	1.2	2.4e-06
**LINC02776**	423	3.5	3.2e-10	958	2.2	1.1e-03
**HIGD2B**	427	3.8	3.2e-10	652	2.6	1.6e-04
**ENSG00000289159.1**	435	3.9	3.8e-10	1,056	2.2	1.8e-03
**ENSG00000264739.1**	438	3.7	3.9e-10	193	3.0	1.1e-06
**ENSG00000267212.1**	447	2.9	4.3e-10	717	2.0	2.5e-04
**ENSG00000273141.1**	458	3.2	5.6e-10	344	2.3	1.1e-05
**ENSG00000279198.1**	461	2.1	5.8e-10	489	1.4	4.7e-05
**ENSG00000287070.1**	472	3.7	7.6e-10	555	2.1	8.5e-05
**ENSG00000289067.1**	473	2.3	7.6e-10	684	1.8	2.0e-04
**LINC01126**	478	2.2	8.2e-10	985	1.5	1.3e-03
**LINC01089**	484	1.2	9.0e-10	901	0.8	8.3e-04
**UAP1-DT**	487	4.7	9.5e-10	397	3.3	1.8e-05
**ENSG00000288919.1**	491	2.6	1.0e-09	1,461	1.1	1.3e-02
**ENSG00000272906.1**	497	2.6	1.1e-09	564	1.5	9.1e-05
**ENSG00000287654.1**	498	3.3	1.1e-09	543	2.5	7.5e-05
**ENSG00000289334.1**	500	2.4	1.1e-09	9	2.5	3.6e-10
**ENSG00000276744.1**	509	3.1	1.2e-09	181	2.9	8.5e-07
**ENSG00000289253.1**	515	2.7	1.3e-09	239	2.6	2.7e-06
**ENSG00000279529.1**	517	1.1	1.3e-09	1,305	0.8	6.7e-03
**ENSG00000288737.1**	532	3.4	1.5e-09	1,153	2.2	3.0e-03
**ENSG00000255089.1**	542	2.9	1.8e-09	600	2.7	1.1e-04
**C10ORF95**	551	3.1	2.0e-09	1,678	1.2	2.5e-02
**ENSG00000274251.1**	552	3.4	2.1e-09	310	2.7	6.5e-06
**ENSG00000270012.1**	560	1.5	2.4e-09	558	0.9	8.7e-05
**ENSG00000274213.1**	615	2.6	6.2e-09	1,908	1.2	4.3e-02
**MIR5188**	618	4.1	6.2e-09	484	3.1	4.4e-05
**ENSG00000288744.1**	630	3.2	7.5e-09	818	1.9	4.3e-04
**ENSG00000272768.1**	663	1.3	1.1e-08	585	1.0	9.7e-05
**ENSG00000289182.1**	664	1.7	1.1e-08	1,386	1.1	9.3e-03
**ENSG00000288939.1**	677	2.7	1.4e-08	646	2.1	1.5e-04
**ENSG00000260651.1**	683	3.5	1.4e-08	1,304	2.1	6.7e-03
**ENSG00000272948.2**	693	2.3	1.5e-08	194	2.1	1.2e-06
**ENSG00000279259.1**	698	2.1	1.6e-08	952	1.3	1.1e-03
**OTULIN-DT**	723	1.8	2.5e-08	619	1.7	1.2e-04
**LINC00677**	726	3.1	2.7e-08	1,273	1.7	5.9e-03
**MIR23AHG**	730	1.7	2.8e-08	517	1.1	6.1e-05
**TIMMDC1-DT**	733	3.1	2.9e-08	298	2.7	5.9e-06
**ENSG00000275709.1**	736	3.0	3.2e-08	447	2.4	2.8e-05
**ENSG00000268670.1**	737	2.0	3.2e-08	1,448	0.9	1.2e-02
**ENSG00000289379.1**	762	3.0	4.3e-08	242	2.5	2.9e-06
**ENSG00000288746.1**	779	2.3	5.6e-08	147	1.9	3.7e-07
**ENSG00000289551.1**	783	3.1	5.8e-08	250	2.9	3.5e-06
**RENO1**	791	1.1	6.4e-08	403	1.1	1.9e-05
**ENSG00000289202.1**	797	3.7	7.0e-08	292	3.1	5.5e-06
**ENSG00000270019.1**	809	2.4	8.1e-08	68	2.6	3.5e-08
**SDR42E2**	873	2.6	2.0e-07	657	2.1	1.6e-04
**RN7SL521P**	877	2.3	2.0e-07	945	1.7	1.1e-03
**ENSG00000273289.1**	914	2.6	2.8e-07	236	2.2	2.6e-06
**ENSG00000289177.1**	918	2.0	2.9e-07	1,019	1.2	1.5e-03
**ENSG00000272969.1**	927	2.9	3.3e-07	824	2.4	4.6e-04
**CPEB2-DT**	934	3.0	3.5e-07	1,066	2.3	1.9e-03
**ENSG00000273363.1**	1,013	2.1	7.5e-07	777	2.1	3.4e-04
**LINC00115**	1,015	1.6	7.6e-07	380	1.4	1.7e-05
**ENSG00000288772.1**	1,020	2.1	8.2e-07	574	1.9	9.3e-05
**ENSG00000280152.1**	1,037	1.6	1.0e-06	589	1.0	9.9e-05
**ENSG00000278546.1**	1,046	1.8	1.1e-06	1,104	1.4	2.3e-03
**ENSG00000288813.1**	1,121	2.3	2.3e-06	1,285	1.8	6.3e-03
**ENSG00000277383.1**	1,126	2.2	2.4e-06	562	1.7	9.0e-05
**ENSG00000279140.1**	1,183	2.1	4.1e-06	1,978	1.1	5.1e-02

	PM/Scl-specific genes at 72h			PM/Scl-specific genes at 24h		
**Gene**	**Pos**	**logFC**	**adj.P.Val**	**Pos**	**logFC**	**adj.P.Val**
**ENSG00000283959.2**	1,215	1.4	5.5e-06	359	1.4	1.4e-05
**ENSG00000288879.1**	1,226	3.8	6.2e-06	1,064	2.8	1.9e-03
**CPNE2-DT**	1,228	2.0	6.3e-06	1,003	1.5	1.4e-03
**ENSG00000279691.1**	1,243	2.7	6.8e-06	1,120	2.2	2.5e-03
**ENSG00000289229.1**	1,259	2.6	7.5e-06	690	2.4	2.0e-04
**ENSG00000273335.1**	1,271	2.1	8.6e-06	1,046	1.5	1.7e-03
**MIR3188**	1,298	2.8	1.1e-05	2,197	1.5	7.3e-02
**ANKH-DT**	1,365	2.3	1.8e-05	2,750	1.5	1.3e-01
**ENSG00000289152.1**	1,417	2.5	3.0e-05	2,711	1.4	1.3e-01
**ENSG00000288963.1**	1,427	1.8	3.2e-05	8,647	0.8	4.9e-01
**ENSG00000274737.1**	1,438	1.9	3.4e-05	1,291	1.1	6.4e-03
**ENSG00000289005.1**	1,456	2.5	3.9e-05	997	2.1	1.4e-03
**DYNLL2-DT**	1,510	2.7	6.2e-05	431	2.3	2.3e-05
**ENSG00000289241.1**	1,594	2.3	1.2e-04	664	2.3	1.7e-04
**ENSG00000262412.1**	1,880	2.1	8.1e-04	1,117	2.1	2.5e-03
**ENSG00000286113.1**	1,896	1.7	8.9e-04	5,335	0.8	3.3e-01
**SMG1-DT**	1,915	2.0	1.0e-03	1,626	1.3	2.1e-02
**ENSG00000289478.1**	2,008	1.9	1.5e-03	1,935	1.6	4.6e-02
**ENSG00000282936.2**	2,325	0.8	5.9e-03	10,801	0.2	5.9e-01
**KLF2-DT**	2,450	1.6	8.2e-03	9,096	0.7	5.1e-01
**LINC00896**	2,798	1.6	1.8e-02	6,855	0.9	4.1e-01
**ENSG00000241666.2**	2,813	1.5	1.9e-02	2,525	1.3	1.1e-01
**ENSG00000289235.1**	3,427	1.5	4.6e-02	1,208	1.9	4.3e-03
**ENSG00000288842.1**	3,515	1.0	5.1e-02	1,633	1.4	2.1e-02
**ENSG00000257258.2**	5,979	0.9	1.8e-01	815	1.6	4.3e-04
**EGFL7**	5,984	-0.6	1.8e-01	12,197	-0.3	6.4e-01
**FSCN1**	6,851	-0.3	2.2e-01	7,539	-0.2	4.4e-01
**ENSG00000273248.1**	7,276	1.0	2.4e-01	3,532	1.2	2.1e-01
**MAP3K11**	7,914	-0.2	2.8e-01	11,507	-0.1	6.2e-01
**CETP**	8,760	0.7	3.2e-01	6,756	0.6	4.0e-01
**EXOC3L2**	10,122	-0.8	3.9e-01	13,950	-0.7	7.1e-01
**RABEP2**	10,627	-0.2	4.1e-01	11,798	-0.1	6.3e-01
**COL18A1**	12,710	-0.3	5.2e-01	21,116	-0.1	9.1e-01
**ENSG00000278158.1**	12,937	0.6	5.3e-01	6,661	0.8	4.0e-01
**TNFAIP8L1**	13,679	-0.1	5.7e-01	19,181	-0.1	8.7e-01
**ITPKB**	14,815	0.2	6.3e-01	8,470	0.3	4.8e-01
**ENSG00000276570.1**	15,050	0.1	6.3e-01	4,558	0.3	2.8e-01
**MIR1915HG**	19,066	-0.2	8.0e-01	16,649	-0.1	8.0e-01
**OIT3**	19,075	-0.2	8.0e-01	25,019	-0.0	9.9e-01
**TNFRSF4**	21,602	-0.1	8.9e-01	17,070	-0.3	8.1e-01
**ENSG00000284484.1**	22,602	-0.1	9.2e-01	23,718	0.1	9.6e-01
**PRAMEF13**	25,349	0.0	9.9e-01	15,696	0.2	7.6e-01
**TRIM51BP**	25,706	-0.0	1.0e+00	2,296	0.9	8.4e-02
